# Editorial: The significance of mitogenomics in mycology, volume II

**DOI:** 10.3389/fmicb.2023.1344877

**Published:** 2023-12-14

**Authors:** Tomasz Kulik, Anne D. van Diepeningen, Georg Hausner

**Affiliations:** ^1^Department of Botany and Nature Protection, University of Warmia and Mazury in Olsztyn, Olsztyn, Poland; ^2^B.U. Biointeractions and Plant Health, Wageningen Plant Research, Wageningen University and Research, Wageningen, Netherlands; ^3^Department of Microbiology, University of Manitoba, Winnipeg, MB, Canada

**Keywords:** fungi, mitochondrial DNA, mobile introns, evolution, genomics

Mitochondria are essential organelles in most eukaryotic organisms, including obligate aerobic fungi. The mitochondria house essential metabolic pathways and components required for oxidative phosphorylation. Their genomes or “mitogenomes” have limited coding capacity and most genes required for the biogenesis, maintenance, and metabolic activity of mitochondria are encoded within the nuclear genome (Calderone et al., 2015; Zardoya, 2020). Fungal mitogenomes are present in multiple copies and are represented as circular molecules although they may exist as linear concatemers (Valach et al., 2011; Chen and Clark-Walker, 2018) that are compacted into mitochondrial nucleoids (Miyakawa, 2017). In fungi, mitogenomes range from 12.055 to >500 kb (James et al., 2013; Liu et al., 2020); size variation can be due to gene content differences, intron mobility, size variation in intergenic spacers, and in some instances the proliferation of repeats (Liu et al., 2020).

Fungal mitogenomes encode for components needed for translation (small and large ribosomal subunit RNAs, a set of tRNAs) and protein components involved in the oxidative phosphorylation pathway. This includes protein components of Complex I (subunits of NADH dehydrogenase: nad1 to nad6 and nad4L), components of Complex III (cob) and Complex IV (cox1, cox2, and cox3), and some members of Complex V (ATP synthase: atp6, atp8, and usually atp9). Some fungal lineages lost the typical Complex I genes (e.g., members of the Taphrinomycota and Sacharomycetales, Schikora-Tamarit et al., 2021; Wolters et al.) and some fungal mitogenomes lost some of the ATP synthase subunit genes (Déquard-Chablat et al., 2011; Zubaer et al., 2018; Mukhopadhyay et al.). Fungal mitogenomes frequently encode a ribosomal protein (rps3; Freel et al., 2015) and sometimes the RNA (rnpB gene) component for RNaseP (Lang, 2018). Mitogenomes can include orphan genes (genes with unknown function and no homologs) and plasmid derived components (Himmelstrand et al.), and there are records of mitogenomes that encode N-acetyltransferases and amino-transferases (Wai et al., 2019).

Across the Mycota, mitogenomes are variable due to recombination events promoted by repeats and by the presence and activities of mobile elements, such group I and group II introns and intron-encoded proteins (IEPs) (e.g., Aguileta et al., 2014; Repar and Warnecke, 2017; Wu and Hao, 2019; Fonseca et al., 2021). Intron encoded proteins for group I introns tend to be homing endonucleases and for group II introns reverse transcriptase-like proteins; these IEPs catalyze intron mobility to cognate intron-less alleles (Belfort et al., 2002). The IEPs can also be involved in aiding the removal of the self-splicing intron RNAs from precursor transcripts by stabilizing intron RNA folds that are a splicing competent (Prince et al., 2022). Mitochondrial introns are gained and lost, and their impact on gene function and phenotypes are still a subject of debate (Goddard and Burt, 1999; Chatre and Ricchetti, 2014; Rudan et al., 2018).

Mitogenome sequences may provide a source of molecular markers for fungal identification, and core protein coding gene sequences can be applied toward resolving evolutionary relationships (Kanzi et al., 2016; Kulik et al., 2020; Kouvelis et al., 2023; Castrillo et al.; Mukhopadhyay et al.). The studies presented in this collection of articles show that members of the same species show very little sequence variation, and differences are usually due to introns (Himmelstrand et al.). Within a fungal lineage, mitogenome size variation can be quite remarkable but it can be accounted for by intron content (Mukhopadhyay et al.). This may not apply to all fungi, as in members of the Basidiomycota mitogenome, size variation is due to introns, intergenic spacers, and intergenic open reading frames (Himmelstrand et al.). The large number of fungal genomes being generated by various research groups provides a resource from which mitochondrial genomes can be extracted for comparative analysis. This allowed Wolters et al. to study mitogenome diversity across the subphylum Saccharomycotina. By analyzing 353 mitogenomes, the authors noted that intron numbers can be quite variable, and introns are poorly conserved among species and lineages within this subphylum. The authors also noted examples of horizontal transfer of introns. Mitochondrial gene expression is assumed to be regulated at the post-transcriptional level. One aspect of fungal mitochondrial gene expression that is different from most metazoans is the removal of introns from the transcripts that encode products that are essential for translation (rRNAs) and for respiration and energy production (Lipinski et al., 2010; Dujon, 2020). The removal of introns is facilitated by IEPs and various nuclear encoded protein factors (Mukhopadhyay and Hausner, 2021; Prince et al., 2022). Mitochondrial ribogenesis, processing of mitogenome derived transcripts, RNA degradation/turnover, and mRNA translation require many nuclear encoded factors (De Silva et al., 2017; Golik, 2023). The reliance on nuclear elements for mitochondrial gene expression links organellar function with nuclear cues in response to environmental and developmental factors.

Wolters et al. observed that among the various orders within Saccharomycotina, the protein coding genes for Complex I were lost independently in several lineages. The authors propose that variations in the mitochondrially encoded protein genes could be driven by evolutionary pressures at the nuclear level. Mukhopadhyay et al. observed that among members of the Ophiostomatales mitogenomes, there are some biases with regard to intron insertion sites and genes that are more likely to be intron-rich, but that there are no conserved introns (except for mL2450 that encodes for RPS3). If introns are beneficial (Belfort, 2017; Rudan et al., 2018) for fine-tuning mitochondrial gene regulation, this is not based on specific introns, instead the mitogenome intron complement is composed of various introns (located at different sites) that are “functionally” redundant. The reliance on nuclear factors for organellar intron splicing impacts mitonuclear compatibilities (or incompatibilities) and potentially imposes reproductive barriers; therefore, they could be promoting speciation events (Dujon, 2020). There are still many questions that need to be addressed with regard to mitonuclear interactions and the associated “cross talk” that impacts mitogenome sequence diversity and gene expression (Wu et al., 2022). Codon usage or biases for fungal mitochondrial genes have not been explored so far. Li et al. demonstrate that there are indeed synonymous codon preferences, and among the examined *Amanita* species, these appear to be under selection. These findings add one more criterion that can influence mitochondrial protein coding sequence diversity as codon usage may have implications for environmental adaptation of mitochondrial genes related to energy metabolism.

This Research Topic in its second volume presents exciting new findings with regard to fungal mitogenomes and mtDNA gene expression and their utility in resolving taxonomic issues, providing insights into the impact of nuclear mitochondrial interactions, which may in part shape mitochondrial gene evolution.

## Author contributions

TK: Conceptualization, Writing—review & editing. AD: Conceptualization, Writing—review & editing. GH: Conceptualization, Writing—original draft.
